# Near-complete de novo assembly of *Tricholoma bakamatsutake* chromosomes revealed the structural divergence and differentiation of *Tricholoma* genomes

**DOI:** 10.1093/g3journal/jkad198

**Published:** 2023-09-02

**Authors:** Hiroyuki Ichida, Hitoshi Murata, Shin Hatakeyama, Akiyoshi Yamada, Akira Ohta

**Affiliations:** Ion Beam Breeding Group, RIKEN Nishina Center for Accelerator-Based Science, Wako, Saitama 351-0198, Japan; Forestry and Forest Products Research Institute (FFPRI), Forest Research and Management Organization, Tsukuba, Ibaraki 305-8687, Japan; Department of Regulatory Biology, Faculty of Science, Saitama University, Saitama, Saitama 338-8570, Japan; Faculty of Agriculture, Shinshu University, Minami-minowa, Nagano 399-4598, Japan; Kansai Research Center, FFPRI, Kyoto, Kyoto 612-0855, Japan

**Keywords:** chromosome-scale assembly, genome evolution, *matsutake* mushroom, phylogeny, *Tricholoma bakamatsutake*, *Tricholoma* section *Caligata*

## Abstract

*Tricholoma bakamatsutake*, which is an edible ectomycorrhizal fungus associated with Fagaceae trees, may have diverged before the other species in *Tricholoma* section *Caligata*. We generated a highly contiguous whole-genome sequence for *T. bakamatsutake* SF-Tf05 isolated in an Oak (*Quercus salicina*) forest in Japan. The assembly of high-fidelity long reads, with a median read length of 12.3 kb, resulted in 13 chromosome-sized contigs comprising 142,068,211 bases with an average guanine and cytosine (GC) content of 43.94%. The 13 chromosomes were predicted to encode 11,060 genes. A contig (122,566 bases) presumably containing the whole circular mitochondrial genome was also recovered. The chromosome-wide comparison of *T. bakamatsutake* and *Tricholoma matsutake* (TMA_r1.0) indicated that the basic number of chromosomes (13) was conserved, but the structures of the corresponding chromosomes diverged, with multiple inversions and translocations. Gene conservation and cluster analyses revealed at least 3 phylogenetic clades in *Tricholoma* section *Caligata*. Specifically, all *T. bakamatsutake* strains belonged to the “*bakamatsutake*” clade, which is most proximal to the “*caligatum*” clade consisting of *Tricholoma caligatum* and *Tricholoma fulvocastaneum*. The constructed highly contiguous nearly telomere-to-telomere genome sequence of a *T. bakamatsutake* isolate will serve as a fundamental resource for future research on the evolution and differentiation of *Tricholoma* species.

## Introduction

*Tricholoma* is a monophyletic genus comprising ectomycorrhizal fungi within the family *Tricholomataceae* (Moncalvo *et al*. 2002). Of those, members of the *Tricholoma* section *Caligata* are ectomycorrhizal symbionts, mainly associated with trees of the families Pinaceae and Fagaceae, and are widely distributed in the temperate and subalpine climatic zones of the Northern Hemisphere (Christensen and Heilmann-Clausen 2013; Heilmann-Clausen *et al*. 2017; Murata, Yamada, *et al*. 2023). *Tricholoma matsutake* and its allied species *Tricholoma bakamatsutake* are valuable edible mushrooms, often traded comprehensively as “*matsutake*,” which grow in Pinaceae and Fagaceae forests, respectively (Supplementary Fig. 1). Because the members of *Tricholoma* section *Caligata* have limited micromorphological variations, they were historically often classified in the wrong taxa and later reclassified according to molecular analyses (Trudell *et al*. 2017; Aoki *et al*. 2022).

*T. bakamatsutake* is a Fagaceae-associated mycorrhizal symbiont that often colonizes the A_0_ soil layer containing litter in both deciduous and evergreen broad-leaved forests, including those with trees from the genera *Quercus*, *Castanopsis*, and *Pasania*, on which fruiting body occurs (Ogawa 1978; Ogawa and Ohara 1978; Terashima 1993; Terashima *et al*. 1993; Herrera *et al*. 2022). Unlike *T. bakamatsutake*, *T. matsutake* is a mycorrhizal symbiont that mainly associates with Pinaceae trees, such as those belonging to the genera *Pinus*, *Tsuga*, *Picea*, and *Abies*, forming a mycelial colony known as “*shiro*” in the B soil layer, which consists mainly of minerals and rocks with relatively few organic compounds (Yamada *et al*. 2006; Yamada *et al*. 2014; Endo *et al*. 2015). In addition to this ecological difference, *T. bakamatsutake* has a genetic and/or epigenetic trait that distinguishes it from *T. matsutake*. More specifically, it often produces phenotypic variants on agar plates, especially after a series of subcultures, changing from a slow-growing brown mycelium to a fast-growing white mycelium. The former produces many chlamydospores with thick hyphae, whereas the latter produces relatively few chlamydospores with thin hyphae on agar plates (Murata, Nakamura, *et al*. 2023). On the basis of genomic analyses of other Agaricomycetes, including the model ectomycorrhizal symbiont *Laccaria bicolor*, ectomycorrhizal fungi may have evolved later than saprophytic fungi (Labbé *et al*. 2012). There has been considerable interest in the evolution and differentiation of *Tricholoma* section *Caligata* in terms of the adaptations to different environments as well as the industrial utility of uncultivated prized mushrooms.

Retrotransposons are one of the most abundant repeating elements in the genomes of eukaryotic organisms, including plant-associated filamentous fungi (Dobinson and Hamer 1993; Spanu *et al*. 2010; Labbé *et al*. 2012). Under the influence of environmental stimuli, retrotransposons replicate through RNA-mediated mechanisms, and their DNA copies are ectopically integrated into the genome, leaving evolutionary footprints on the genome. In *Tricholoma* section *Caligata*, 3 types of full-length retrotransposons have been identified, namely *marY1*, *marY2N*, and *megB1*, of which, the first 2 are thus far exclusive to this taxon (Murata *et al*. 1999, 2001; Murata and Yamada 2000; Murata, Ota, Yamaguchi, *et al*. 2013). In addition, *marY1* is approximately 6 kb long and includes a 426-bp long terminal repeat (LTR) designated as *σ_marY1_*, which structurally resembles mammalian retroviruses. Moreover, it is transcribed and undergoes postreplication transposition (Murata and Yamada 2000; Murata and Miyazaki 2001, 2004). In contrast, *marY2N* is a long interspersed nuclear element (LINE) that structurally resembles mRNA with a poly-(A) tail (Murata *et al*. 2001), and *megB1* is a dimeric *Alu*-like element (e.g. *AbaMEG1–AbaMEG2*). Earlier research showed that *Alu* is a short mobile DNA sequence with no coding region to replicate and integrate and is abundant in the form of a dimer in humans and primates (Deininger 1989; Kazazian 2000). Many Agaricomycetes fungi carry *megB1* in rRNA gene intergenic spacer 1 (IGS1) as the main component and multicopy element, but the fungi belonging to *Tricholoma* section *Caligata* carry a copy of *megB1* outside of the rRNA gene (Babasaki *et al*. 2007). On the basis of the copy numbers of these retrotransposons and the short repeating sequences surrounding *magB1*, the diversification of the species in *Tricholoma* section *Caligata* may have occurred as follows: *T. bakamatsutake* differentiated first, followed by Fagaceae-associated *Tricholoma fulvocastaneum* and then *T. matsutake* (Murata, Ota, Yamaguchi, *et al*. 2013).

The genome structures and sequences of most *Tricholoma* species have not been elucidated, but there have been several recent attempts at determining the *T. matsutake* whole-genome sequence (Min *et al*. 2020; Miyauchi *et al*. 2020). A telomere-to-telomere *T. matsutake* genome sequence was reported, but the sequencing was performed on 2 fruiting bodies collected from the wild and the sequenced fungus is not available as a strain (TMA_r1.0; Kurokochi *et al*. 2023). There is little available genomic information regarding the phenotypically unstable *T. bakamatsutake*, which has ecological traits distinct from those of *T. matsutake* and may have diverged earlier than many other species belonging to the *Tricholoma* section *Caligata* (Murata, Ota, Yamaguchi, *et al*. 2013). In the present study, we performed a whole-genome sequencing analysis of *T. bakamatsutake* strain SF-Tf05, which was originally collected in Shiga, Japan, and has been stably maintained on agar for more than 10 years without any observable morphological changes (Murata, Nakamura, *et al*. 2023). We constructed a nearly complete and telomere-to-telomere assembly of the strain using highly accurate long-read HiFi sequencing technology (Pacific Biosciences, Menlo Park, CA, USA). A comparative genomic study using the resulting chromosome-sized assembly revealed that the chromosomal structure and sequence of the centromeric regions in *T. bakamatsutake* are clearly different from those of its close relative, *T. matsutake*. The high-quality *T. bakamatsutake* SF-Tf05 genome sequence will serve as the foundation for future investigations on the evolution and differentiation of *Tricholoma* section *Caligata* and its related taxa.

## Materials and methods

### Fungal strains and growth conditions

The fungal strains used in this study are listed in Table 1. Unless otherwise stated, mycelia were cultured at 23°C on modified Melin–Norkrans (MMN) medium containing 1.5% V8 juice (MMN + V8; Murata *et al*. 1999). For the extraction of high molecular weight genomic DNA, fungal mycelia were precultured on semisolid MMN + V8 agar plate with 0.3% SeaKem GTG agarose, cut into small pieces with a surgical scalpel, and inoculated into 100 mL of MMN + V8 liquid medium prepared in 4–6 1-L Erlenmeyer flasks sealed with cotton plugs. The liquid cultures were placed on shelves in an incubation room with vigorous horizontal agitation for approximately 20 s, 3 times a day. Mycelia were grown to the onset of the exponential phase, which took approximately 2–6 weeks depending on taxa and strains, and then collected with nylon mesh filters and stored at −80°C.

**Table 1.** Fungal strains used in this study.

Species*^a^*	Strain	Sampling site and vegetation	Year isolated	Reference*^b^*
*Tricholoma anatolicum*	TK S-2-2	*Cedrus libani* forest, Gal Mountain, Turkey	2006	1, 2, 3
	MC1 (ATCC MYA-929)*^c^*	Kingdom of Morocco	1998	1, 2, 3
*Tricholoma bakamatsutake*	SF-Tf05	*Quercus salicina* forest, Shiga, Japan	2005	1, 2, 4
	SF-Tf08	*Quercus salicina* forest, Shiga, Japan	2005	5
	SF-Tf09	*Quercus salicina* forest, Shiga, Japan	2005	1, 4
	B1 (NBRC 33138)*^d^*	*Quercus serrata* forest, Ibaraki, Japan	1993	3, 7, 8
	CB-Tb1 (NBRC 108265)	*Pasania edulis/Castanopsis sieboldii* forest, Chiba, Japan	1989	6
	NF 3028*^e^*	Mixed forest of *Quercus dentata*, *Quercus serrata*, *Quercus crispula*, Iwate, Japan	2010	9
	NF 3036*^e^*	Mixed forest of *Quercus dentata*, *Quercus serrata*, *Quercus crispula*, Hokkaido, Japan	2010	9
	NF 3042*^e^*	*Quercus glauca* at a roadside, Oita, Japan	2010	9
	W147	Wakayama, Japan	1989	9
	EH-Tb1	Ehime, Japan	1987	9
*Tricholoma caligatum*	R106 (NBRC 109035)	*Pinus pinea* forest, Carabria, Italy	2011	1, 2, 5
	R107 (NBRC 109036)	*Pinus pinea* forest, Carabria, Italy	2011	1, 2, 5
*Tricholoma fulvocastaneum*	LAOS 1	Laos	2011	5
	WK-N-1 (ATCC MYA-922, NBRC 108268)	*Quercus phillyraeoides* forest, Wakayama, Japan	1988	5, 6
*Tricholoma matsutake*	Y1*^f^* (ATCC MYA-915, NBRC 33136)	*Pinus densiflora* forest, Ibaraki, Japan	1993	1–3, 5–8
	AT925	Sweden	2000	3, 8
	H1	Bhutan	1998	6
*Tricholoma mesoamericanum*	MX1 (ATCC MYA-921)	Mexico (originally described as *Tricholoma matsutake*)	1998	1–3, 5, 6
	TM-4	Mexico (originally described as *Tricholoma matsutake*)	1992	1, 2, 3, 5
*Tricholoma murrillianum*	TM-10	Canada	1992	1, 2, 5
	Tp-C3 (ATCC MYA-930)	Canada	1994	1, 2, 3, 5
*Tricholoma robustum*	Tr1	Hokkaido, Japan	1966	—
	Tr4 (ATCC 204331)	Shiga, Japan	1990	—

Phylogenetic relationships among the following matsutake species have been reported (Ota *et al*. 2012; Murata, Ota, Yamada, *et al*. 2013; Aoki *et al*. 2022).

References: 1, Ota *et al*. (2012); 2, Murata, Ota, Yamada, *et al*. (2013); 3, Aoki *et al*. (2022); 4, Murata, Nakamura, *et al*. (2023); 5, Murata, Ota, Yamaguchi, *et al*. (2013); 6, Murata *et al*. (1999); 7, Yamada *et al*. (2014); 8, Yamada *et al*. (2010); 9, this study.

American Type Culture Collection, USA.

NITE Biological Resource Collection, National Institute of Technology and Evaluation, Chiba, Japan.

Kindly provided by the Nara Forest Research Institute, Nara, Japan.

This is a model organism that produces a rhizosphere colony that resembles naturally occurring “*shiro*” under in vitro conditions (Yamada *et al*. 2006; Murata, Yamada, *et al*. 2013).

### DNA extraction and sequencing

Genomic DNA was extracted from frozen mycelia using Genomic-tip (Qiagen, Hilden, Germany), with minor modifications to the manufacturer's protocol. Briefly, a 500-mg frozen mycelial mat was ground in liquid nitrogen using a mortar and pestle. The resulting fine powder was resuspended in 5-mL buffer G2. After adding 10-µL RNase A (100 mg/mL, Qiagen) and 250-µL proteinase K (20 mg/mL, Wako Pure Chemical, Osaka, Japan), the sample was incubated for 30 min at 55°C with gentle agitation. Next, 3-mL chloroform was added, and the solution was gently mixed in a rotator for 10 min. After centrifuging at 10,000 × *g* for 10 min at 4°C, the supernatant was applied to a Genomic-tip 100/G and processed according to the manufacturer's protocol. The resulting DNA was resuspended in 1× IDTE buffer (Integrated DNA Technologies, Coralville, IA, USA) and quantified using the QuantiFluor One dsDNA kit (Promega, Madison, WI, USA). The HiFi reads were obtained using the SMRT Cell 8M and the PacBio Sequel II instrument, which was operated in the circular consensus sequencing mode (Pacific Biosciences). The library was constructed and sequenced at the Genomics and Cell Characterization Core Facility (GC3F) at the University of Oregon (Eugene, OR, USA).

### Genome assembly and bioinformatics analysis

The default parameters of all programs were used unless otherwise specified. The HiFi reads were assembled using *hifiasm* (version 0.16.1-r375; Cheng *et al*. 2021). Smaller contigs (i.e. not including the 13 largest contigs) were subjected to a homology search, with BLASTN (version 2.12.0+; Altschul *et al*. 1997) used to screen the *Tricholoma* sequences in GenBank to identify possibly misassembled sequences and mitochondrial contigs. A Benchmarking Universal Single-Copy Orthologs (BUSCO; Simao *et al*. 2015) analysis using the Docker container (version 5.4.3_cv1) was performed to evaluate the completeness of the genome assembly. Telomere regions were identified by a BLASTN search involving 5 tandem repeats of the reported fungal 6-mer telomeric repeating unit (5′-CCCTAA-3′; Červenák *et al*. 2020; Rahnama *et al*. 2021), with an *E*-value cutoff of 1.0e^−10^. The *nucmer* program in the MUMmer4 suite (Marcais *et al*. 2018) was used for comparing genomes. The matching regions were filtered using the “-l 10000” option of the *delta-filter* program and plotted using Gnuplot (version 5.4 patchlevel 2; available from http://www.gnuplot.info).

Chromosomal genes were predicted using the FunGAP pipeline (version 1.1.1; Min *et al*. 2017) with RNA-seq reads from *T. matsutake* 945 deposited in the Sequence Read Archive (accession code PRJNA200596; last updated on 2020 October 20). The predicted genes were annotated according to a homology search of the NCBI nonredundant protein (nr) database (downloaded from https://ftp.ncbi.nlm.nih.gov/blast/db/FASTA, on 2022 September 25) using the following options of the DIAMOND program (Buchfink *et al*. 2021): “–evalue 1e-10 –more-sensitive --query-cover 50 –subject-cover 50”. To functionally annotate genes, the KEGG Automatic Annotation Server (KAAS; https://www.genome.jp/kegg/kaas) was used along with an SBH method, GHOSTZ for the homology search, and the GENES data set for 40 species (hsa, mmu, rno, dre, dme, cel, ath, sce, ago, cal, spo, ecu, pfa, cho, ehi, eco, nme, hpy, bsu, lla, mge, mtu, syn, aae, mja, ape, fox, mgr, ncr, bfu, ani, aor, ang, tms, ppl, tvs, hir, mpr, scm, and uma). The mitochondrial genome was annotated using MITOS2 (Donath *et al*. 2019), with “RefSeq 89 Fungi” as the reference and the “Mold” genetic codes.

### Repeated sequence and syntenic analyses

Transposable elements and other repeated sequences were identified ab initio using RepeatModeler2 (version 2.0.3; Flynn *et al*. 2020), with the “-LTRStruct” option. Repetitive sequences within the chromosome assembly were identified using RepeatMasker (version 4.1.2-p1; developed by Smit, A.F.A., Hubley, R., and Green, P., downloaded from https://www.repeatmasker.org/), with the “-xsmall” option. Repeats were classified on the basis of the RepeatMasker output. The genomic distributions of *marY1* (GenBank accession code AB028236) and *marY2N* (AB047280) were determined using RepeatMasker and FASTA-formatted sequences as the repeat libraries. The LINE, *marY2N*, and LTR contents were calculated as the percentage of the masked regions in 5- (LINE and *marY2N*) or 50-kb (LTR and *marY1*) windows and plotted using Gnuplot. The guanine and cytosine (GC) content of genomic regions was calculated using GCcalc.py (version 85b6ab5; downloaded from https://github.com/WenchaoLin/GCcalc), with window and step sizes of 50 kb, and plotted using Gnuplot. The resulting plots were combined into a figure using Adobe Illustrator CS6 (Adobe Inc., San Jose, CA, USA). Genes within nonrepetitive regions were extracted using the “intersect” tool in BEDTools (Quinlan and Hall 2010) and the Tbkm_v1 repeat library created with RepeatModeler. The corresponding gene regions between Tbkm_v1 and TMA_r1.0 (Kurokochi *et al*. 2023) were determined using TBLASTN, with an *E*-value cutoff of 1.0e^−10^. Hits with the highest bit score and lowest *E*-value were considered the corresponding regions between these assemblies and visualized using the “Advanced Circos’ tool in the TBtools program (Chen *et al*. 2020).

### Gene conservation and mapping analyses of Tbkm_v1 and TMA_r1.0

The gene regions predicted by the FunGAP program were used as the input for the gene conservation analysis. Short-read sequences from different *Tricholoma* species were mapped to Tbkm_v1 and TMA_r1.0 as previously described. The resulting BAM files were used for the gene coverage analysis. Mapping rates were calculated using the “idxstat” tool in SAMtools (Danecek *et al*. 2021). We used a combination of in-house C++ programs to calculate the gene coverage in the 2 reference sequences for each sample. First, read depth matrices at each chromosomal position were created using the “*create_read_count_matrix*” program, and then the percentage of gene regions that were covered with 3 or more reads was calculated using the “*gff_coverage*” program. The resulting gene coverage information for Tbkm_v1 and TMA_r1.0 was combined and subjected to a cluster analysis using the “heatmap” function in the R statistical environment (R Core Team 2002).

## Results and discussion

### Nearly telomere-to-telomere assembly of *T. bakamatsutake* SF-Tf05 chromosomes

**Fig. 1.**
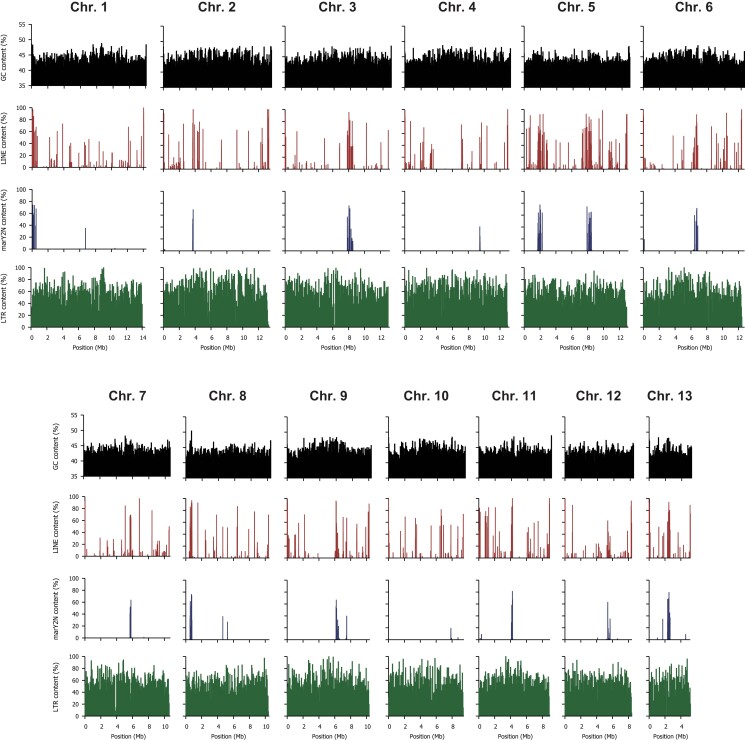
Characteristics of *T. bakamatsutake* SF-Tf05 chromosomes. The contents of GC nucleotides, LINEs, *marY2N*, and LTR-type retrotransposons for different chromosomes are presented. The window sizes are 50 kb for GC, LINE, and LTR and 5 kb for *marY2N*. Unlike in *T. matsutake*, GC- and LINE-rich regions were not detected in the chromosomal regions.

It is well known that fungi belonging to *Tricholoma* section *Caligata* grow very slowly, making it difficult to obtain high-purity genomic DNA necessary for genome sequencing. We found that mycelia in early exponential phase in liquid medium, inoculated with precultured mycelia on semisolid plates, is suitable to extract high molecular weight genomic DNA with less impurities. The extracted DNA contained a detectable amount of smaller fragments by agarose gel electrophoresis (Supplementary Fig. 2), presumably due to autodegradation in dead mycelial cells, since this strain grows very slowly (4–6 weeks for the exponential growth phase) to maintain genome integrity against hyphal senescence. A sequencing run with the SMRT Cell 8M and the PacBio Sequel II instrument yielded 30.24 Gb of highly accurate long reads (HiFi reads) with a minimum quality of Q_20_ (i.e. consensus base call accuracy of 99%) and a median read length (read N_50_) of 12,294 bp. The assembly statistics are provided in Table 2. The assembly of the genome using *hifiasm* (Cheng *et al*. 2021) resulted in a total of 201 contigs with a total length of 148.26 Mb and a contig N_50_ value of 12.43 Mb. The longest contig was 14.07 Mb. The 13 longest contigs represented 95.82% (142.07 Mb) of the total contig length. The lengths of the 13th and 14th contigs differed by more than 10 times (5.23 and 0.31 Mb, respectively). Therefore, we considered the 13 longest contigs as the primary contigs and designated them as chromosomes 1–13 in descending order of their nucleotide length. The BLASTN search using the reported telomeric repeat sequence in fungi (5′-CCCTAA-3′; Červenák *et al*. 2020; Rahnama *et al*. 2021) revealed telomeric repeats on at least 1 side of all chromosomes. Nearly half of the *T. bakamatsutake* chromosomes (chromosomes 1, 4, 7, 9, 10, and 12) had telomeric repeats at both ends, indicating that most of the assembled sequences were telomere-to-telomere sequences. The assembled chromosome lengths and GC contents varied from 14.07 to 5.23 Mb and from 43.39 to 44.52%, respectively (Supplementary Table 1). The overall GC content of the 13 chromosomes was 43.94%, which was similar to the GC contents of previously reported *T. matsutake* genome assemblies, with an average of 45.39% in 3 different assemblies (Min *et al*. 2020; Miyauchi *et al*. 2020; Kurokochi *et al*. 2023). Unlike the *T. matsutake* chromosomes, most of the *T. bakamatsutake* chromosomal regions had similar GC contents. The exceptions were chromosomes 2 and 8, which had a GC-rich region near one of the ends. These observations suggested the physical structures of the centromeric regions may differ between these 2 species (Fig. 1). The BUSCO analysis indicated that the 13 chromosomes contained 97.7% (741 of 758) of the core genes defined in the fungi_odb10 data set, suggesting the assembly is almost complete. The final chromosomal assembly was designated Tbkm_v1 and deposited in GenBank with the accession codes CP114857–CP114869 (chr01–13).

**Table 2.** Statistics for the Tbkm_v1 assembly.

Assembly	All contigs	Tbkm_v1	Mitochondrial genome (mt)
# contigs	201	13	1
Total length (bp)	148,260,793	142,068,211	122,566
Largest contig (bp)	14,073,764	14,073,764	n.a.
GC content (%)	43.90	43.94	21.70
N_50_	12,427,164	12,427,164	n.a.
N_75_	9,419,585	10,325,240	n.a.
L_50_	6	6	n.a.
L_75_	10	9	n.a.
Ns per 100 kb	0	0	0

n.a., not applicable.

### Prediction of chromosomal genes

Protein-coding genes on chromosomes were predicted using the FunGAP2 pipeline (Min *et al*. 2017) and RNA-seq reads from 2 different analyses of *T. matsutake* (SRA BioProject codes PRJNA365663 and PRJNA536102). Although this was a heterologous mapping between different species, the mapping rate of the RNA-seq reads to the Tbkm_v1 assembly was 63.16% (a total of 314.07 million mapped reads), which was sufficient for creating gene models on the basis of the conserved genes. The FunGAP pipeline predicted 11,060 genes encoded by the 13 chromosomes, with an average gene density of 1 per 13.42 kb (range: 1 gene per 9.89–16.69 kb among chromosomes). The predicted proteins covered 96.7% (733 of 758) of the BUSCO groups defined in the fungi_odb10 data set, reflecting the suitability of the method used for predicting genes. The predicted genes were annotated by using the deduced amino acid sequences as queries to screen the nr database for similar sequences, with a cutoff threshold of 50% coverage in both query and hit sequences. Of the 11,060 predicted gene products, 9,200 (83.18%) had a significant hit in the nr database. Approximately 80% (7,512 gene products; 83.18%) of these best hits in the nr database were predicted proteins in *T. matsutake* 945. Additionally, nearly 60% (5,416 gene products; 58.87%) were annotated as “hypothetical protein,” “uncharacterized protein,” and “unnamed protein product.” Although the remaining gene products also had unclear functions, they were mostly similar to cytochrome P450 (77 proteins), major facilitator superfamily (MFS) general substrate transporters (74 proteins), and alpha/beta-hydrolases (58 proteins). The KAAS analysis mapped 4,802 predicted genes to the KEGG Orthology (KO) groups and assigned the genes to functional categories and biological pathways. Supplementary Table 2 lists the number of genes in each functional group. In addition to the genes assigned to groups associated with housekeeping functions, such as membrane trafficking (ko04131; 411 genes) and messenger RNA biogenesis (ko03019, 204 genes), many genes were classified as transporters (ko02000, 209 genes), peptidases and inhibitors (ko01002, 149 genes), and protein kinases (ko01001, 119 genes). A total of 365 tRNA genes were identified by the tRNAscan-SE program, whereas another 7 genes were classified as “tRNAs with undetermined/unknown isotypes.” Interestingly, Tbkm_v1 included more than the expected number of tRNA-Ile genes, most of which (275 of 277) harbored the TAT anticodon.

### Mitochondrial genome

The BLASTN searches using the remaining 188 contigs to screen the NCBI nucleotide database (nr/nt) revealed a contig (138,202 bp) that matched a previously reported complete mitochondrial genome sequence in *T. bakamatsutake* (GenBank accession code MN873035; 103,090 bp). The 2 mitochondrial genome sequences were highly similar (i.e. sequence identity exceeding 99%). The sequence comparison showed that our initially assembled mitochondrial sequence had overlapping sequences at each end, with the first 15,636 bp perfectly matching the sequence at the 3′ end. This overlap indicated that the mitochondrial genome was organized as a closed master circle. One of the 2 duplicated regions was manually removed. The remaining 122,566-bp sequence was designated as “*mt*” and considered to represent the complete *T. bakamatsutake* SF-Tf05 mitochondrial genome sequence. Although the assembled *mt* sequence was highly similar to a previously reported mitochondrial genome sequence (MN873035; Huang *et al*. 2021), there were 5 major differences: an inversion between positions 9,623 and 22,404 (12,782 bp) as well as 4 insertions between positions 7,573 and 9,622 (2,050 bp), 22,405 and 31,465 (9,061 bp), 63,476 and 64,161 (686 bp), and 81,405 and 88,967 (7,563 bp). The Liftoff program (Shumate and Salzberg 2020) mapped all of the 228 annotated features in MN873035, of which 213 were detected as single copies and the remaining 15 had an extra copy in the *mt* sequence. There were 81 protein-coding genes and 25 genes encoding tRNAs corresponding to 17 of the 20 amino acids in the SF-Tf05 mitochondrial genome. The sequence analysis indicated that the SF-Tf05 mitochondrial genome lacks tRNA genes for proline (AGG, GGG, CGG, and TGG), glutamic acid (CTC and TTC), and cysteine (ACA and GCA). The same tRNA gene analyses were also performed for MN873035 and AP026551, which are the complete mitochondrial genome sequences in *T. bakamatsutake* and *T. matsutake*, respectively. Similar to the results for *T. bakamatsutake* SF-Tf05, the MN873035 and AP026551 sequences did not include tRNA genes for proline, glutamic acid, and cysteine. In addition to the tRNAs for these 3 amino acids, MN873035 also lacked tRNAs for lysine, serine, and tyrosine. These observations revealed that some tRNA genes in *Tricholoma* species are encoded in the nuclear genome. To further investigate the completeness and possible sequencing and assembly errors in common mitochondrial genes, we performed a de novo gene prediction and annotation using MITOS2 (Donath *et al*. 2019) for both SF-Tf05 and MN873035. The results indicated the coding regions of the *dpo* and *lagli* genes in SF-Tf05 and MN873035 may include stop codons. Some additional stop codons were also detected in the SF-Tf05 *giy* gene. The Sanger sequencing of the PCR-amplified fragments for these regions in SF-Tf05 confirmed the presence of all of these mutations in the genome (data not shown). The resulting complete mitochondrial genome sequence was deposited in GenBank with the accession code CP114870.

### Repeated sequences in the *T. bakamatsutake* genome

The repeated sequences in the Tbkm_v1 assembly were identified using RepeatModeler and RepeatMasker. In the Tbkm_v1 assembly, 78.68% (111.78 Mb) of the entire genome consisted of repetitive sequences. The abundance of each repetitive sequence is summarized in Table 3. The LTR retrotransposons were the most abundant type of repeated sequences in Tbkm_v1 (i.e. 53.16% of the genome). Almost all of the LTR-type elements were classified as Ty3/Gypsy (74.67% of all LTR regions) or Ty1/Copia (19.55%). The other types of repeated sequences in Tbkm_v1 were mostly DNA transposons (6.66% of the genome), rolling circles (6.38%), and other unclassified interspersed repeats (10.82%). The total length of small RNA, simple repeats, and other low-complexity regions represented less than 1% of the genome. Compared with *T. matsutake*, the localization of LINEs was less extensive in Tbkm_v1, with multiple chromosomal regions that contained many LINEs. Moreover, the locations of LINE-rich regions were not highly correlated with the GC contents (Fig. 1). Some of these LINE-rich regions overlapped the localized regions containing *marY2N*-like sequences, which were previously characterized as LINE-like non-LTR retroelements. With the exception of chromosome 10, which did not have an apparent localized peak, the *marY2N*-rich regions were located only near the center or the end of chromosomes. These observations suggest that *marY2N* and its derivatives are enriched in centromeric regions. Another well-studied retroelement in *T. matsutake*, *marY1* (Murata and Yamada 2000; Kurokochi *et al*. 2023), was distributed across all 13 chromosomes in Tbkm_v1 (Supplementary Fig. 3). A BLASTN search using the full-length *marY1* sequence (GenBank accession code AB028236) as the query and an *E*-value cutoff of 1.0e^−10^ detected 559 copies of *marY1*-like sequences in the assembly.

**Table 3.** Repetitive sequences in the Tbkm_v1 assembly.

Type of repeated sequence	Num. of elements	Total length (bp)	Percentage of genome
SINEs	299	81,536	0.06
LINEs	1,736	1,756,689	1.24
LTR elements	58,477	75,528,114	53.16
DNA transposons	10,103	9,464,515	6.66
Rolling circles	4,969	9,030,370	6.36
Unclassified interspersed repeats	44,596	15,366,895	10.82
Small RNA	379	119,893	0.08
Simple repeats	9,742	473,500	0.33
Low complexity	674	40,320	0.03
Total		111,780,296	78.68

### Mating-type (*MAT*) loci in *T. bakamatsutake*

**Fig. 2.**
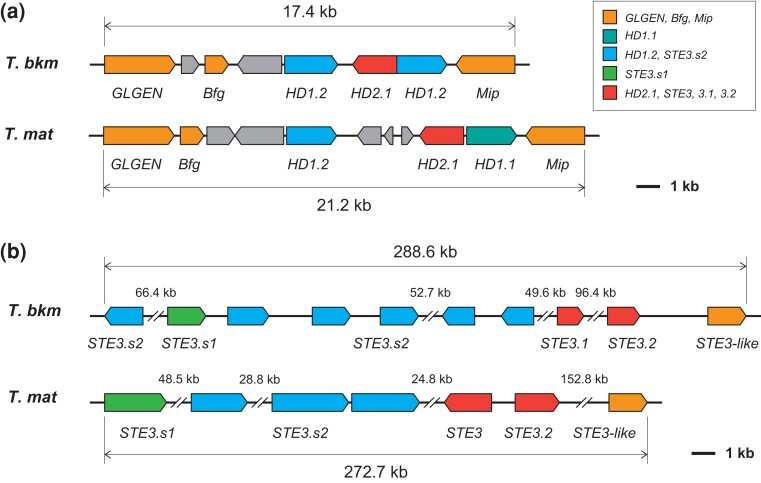
Structures of the mating-type loci in *T. bakamatsutake* and *T. matsutake*. Gene organizations in the probable *MATA* (a) and *MATB* (b) loci in *T. bakamatsutake* (*T. bkm*) and *T. matsutake* (*T. mat*). Box colors reflect grouping by similarity to known mating-type genes. The genetic contents in the *MAT* regions are similar between the 2 *Tricholoma* species.

The mating-type (*MAT*) genes have been extensively studied in many *Basidiomycota* fungi (Kües 2015). The bipolar and tetrapolar systems are the 2 major types of systems that determine the mating type in *Basidiomycota* fungi. The 2 *MAT* loci, *MATA* (syn. *b* or *HD*) and *MATB* (syn. *a* or *P/R*), which comprise a cluster of homeodomain transcription factor genes and pheromone receptor (*STE3*) and precursor (*Phe3*) genes, are linked at a single *MAT* locus (bipolar system) or are separated, often on different chromosomes (tetrapolar system) (Peris *et al*. 2022). Homology searches using the deduced HD and STE3 protein sequences of *Hypsizygus marmoreus* (Wang *et al*. 2021) revealed that *T. bakamatsutake* harbors *HD* and *STE3* orthologs on different chromosomes, implying that the mating type in this species is determined by the tetrapolar system. The schematic structures of the *MATA* and *MATB* loci in *T. bakamatsutake* are presented in Fig. 2. The *MATA* locus (approximately 17.4 kb region on chromosome 2) is flanked by the mitochondrial intermediate peptidase (*Mip*) and beta-flanking (*Bfg*) genes, which is consistent with recent findings regarding *H. marmoreus* (Wang *et al*. 2021). Similar to *L. bicolor*, but unlike the 2 *Trichaptum* species with a sequenced genome and some other *Basidiomycota* fungi (James *et al*. 2004; Niculita-Hirzel *et al*. 2008; Peris *et al*. 2022), the glycogenin-1 (*GLGEN*) gene is located immediately next to *Bfg* in *T. bakamatsutake*. The region between *Mip* and *Bfg* was predicted to include 4 genes, of which the deduced amino acid sequence of *SF02g08880* matched HD2.1 of *H. marmoreus* (QQL12049), whereas *SF02g08870* and *SF02g08890* matched HD1.2 (QQL12048). The *MATB* locus (approximately 288.6-kb region on chromosome 11) contains 6 genes encoding STE3.s2-like proteins (*SF11g01200* and *SF11g01290–SF11g01330*) as well as individual genes encoding STE3.s1-, STE3.1-, and STE3.2-like proteins (*SF11g01280*, *SF11g01340*, and *SF11g01380*, respectively). An additional STE3-like gene (*SF11g01390*) was detected in *T. matsutake* (KAF8222657) and *Lepista nuda* (syn. *Clitocybe nuda*, KAF9468417), an edible mushroom in the family Tricholomataceae, but not in *H. marmoreus*. The structures and gene organizations in the *MATA* and *MATB* loci were essentially conserved in *T. bakamatsutake* and *T. matsutake* (Fig. 2). These results indicated that mating compatibility is determined by a tetrapolar system in *T. bakamatsutake* and *T. matsutake*.

### Common and unique genes in the 2 *Tricholoma* species

**Fig. 3.**
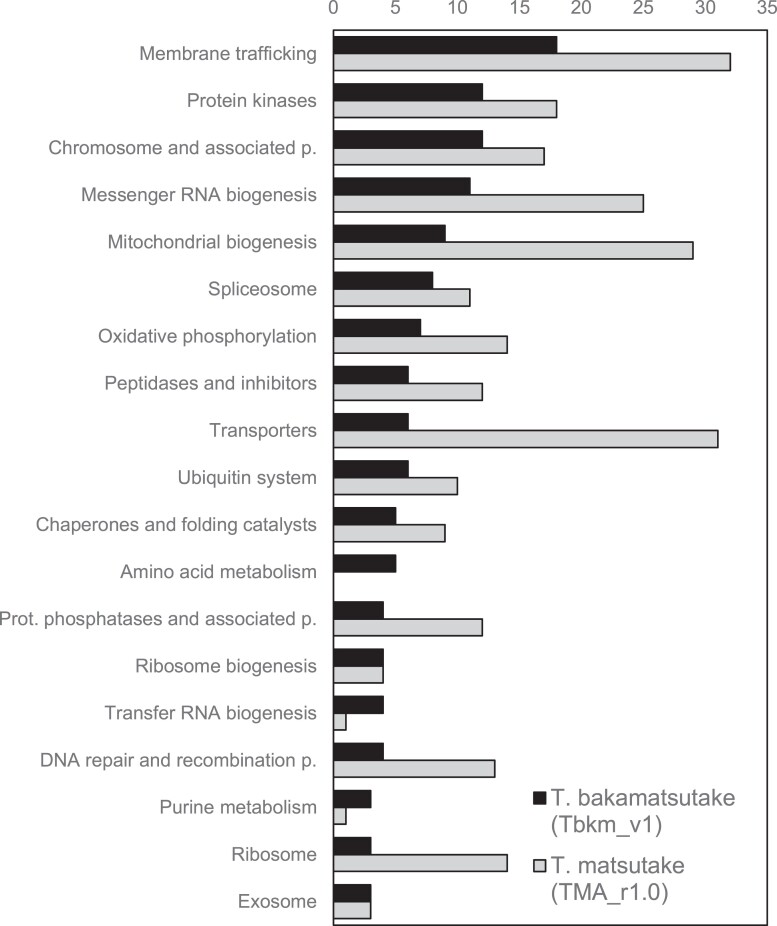
Functional categorization of genes that were present in either *T. bakamatsutake* (Tbkm_v1) or *T. matsutake* (TMA_r1.0). Bars indicate the number of genes in each functional category. Genes were categorized according to the KO database.

We investigated the gene conservation between *T. bakamatsutake* and *T. matsutake* using the chromosome-scale assemblies, Tbkm_v1 and TMA_r1.0. Although TMA_r1.0 was previously predicted to contain 28,322 protein-coding genes, information regarding these genes was not publicly available when we conducted the present study. Therefore, the genes in TMA_r1.0 were predicted and annotated according to the method used for Tbkm_v1. The FunGAP pipeline revealed 18,646 protein-coding genes encoded by the 13 chromosomes in TMA_r1.0. We subsequently used all of the predicted protein sequences from Tbkm_v1 and TMA_r1.0 to cross-search the matching region via a TBLASTN search with an *E*-value cutoff of 1.0e^−10^. There were 928 (8.39% of the predicted genes in Tbkm_v1) and 5,395 (28.94% of our predicted genes in TMA_r1.0) genes that were present in 1 of the 2 assemblies. Another 893 (8.07%) and 2,767 (14.84%) genes had deduced amino acid sequence similarities/identities less than 50% in Tbkm_v1 and TMA_r1.0, respectively. The remaining 9,239 (83.54%) and 10,484 (56.23%) genes were considered to be common to both species. Even though more genes were predicted for TMA_r1.0 than for Tbkm_v1 because RNA-seq reads from the same species were used, it was surprising that nearly 30% of the genes were detected only in TMA_r1.0, while more than 80% of the genes in Tbkm_v1 were present in both species. The functional categories of the genes that were present in at least 1 of the species are presented in Fig. 3. Protein kinases (ko01001; 12 and 18 unique genes in Tbkm_v1 and TMA_r1.0, respectively), peptidases and their inhibitors (ko01002; 6 and 12 genes), transporters (ko02000; 6 and 31 genes), and amino acid metabolism (ko99985; 5 genes in Tbkm_v1) were the notable categories in addition to some of the large functional categories associated with the protein-coding genes in the genomes described above. Many of the species-specific genes were revealed to encode relatively small peptides and proteins: 216.23 ± 205.69 (average ± SD) and 216.67 ± 194.14 in Tbkm_v1 and TMA_r1.0, respectively. These peptides and proteins were smaller than the common gene products in Tbkm_v1 and TMA_r1.0: 458.86 ± 367.83 and 437.81 ± 358.81, respectively.

Multiple *T. bakamatsutake* genes were detected exclusively in Tbkm_v1. The functionally annotated genes included *SF01g07050* (encoding endo-1,4-β-xylanase); *SF12g05070* (encoding cholesterol 7-α-monooxygenase); *SF03g00020* and *SF04g00010* (encoding mitogen-activated kinase-like proteins); *SF01g08930*, *SF01g08940*, *SF01g09180*, *SF06g10950*, and *SF07g06230* (possibly encoding glutamine amidotransferase DUG3-like proteins according to the KAAS results); and 5 genes (*SF01g08930*, *SF01g08940*, *SF01g09180*, *SF06g10950*, and *SF07g06230*) that were significantly similar to the gene encoding the “hypothetical protein H0H93_006617” containing a YafJ-type glutamine amidotransferase-like domain (cd00352 in the NCBI Conserved Domain Database; Yang *et al*. 2020) from the basidiomycete *Arthromyces matolae*. In contrast, genes encoding AMY (α-amylase, *TM03g03410*), PHO (acid phosphatase, *TM01g00540*), and endo-β-glucanase-like (*TM05g15980*) were present only in TMA_r1.0. We determined that Tbkm_v1 lacks a gene encoding PTEN, which catalyzes the dephosphorylation of phosphatidylinositol 3,4,5-trisphosphate (PIP_3_) to produce phosphatidylinositol-4,5-bisphosphate (PIP_2_). Compared with other membrane phospholipids, PIP_2_ and PIP_3_ are less abundant and they function as second messengers in several crucial cellular processes, such as membrane trafficking and cell signaling, and are highly conserved across kingdoms. The phosphatase PTEN contributes to a negative regulatory mechanism that converts PIP_3_ back to PIP_2_, thereby decreasing the abundance of PIP_3_ in the plasma membrane (Czech 2000). Theoretically, a lack of PTEN increases the PIP_3_ content and may lead to the continuous activation and deactivation of downstream processes. Therefore, it is possible that some of the presented genetic diversity was the result of the differences in the adaptations to environmental conditions between *T. bakamatsutake* and *T. matsutake*, which establish ectomycorrhizal symbiotic relationships with broad-leaved deciduous Fagaceae trees and coniferous Pinaceae trees, respectively.

### *T. bakamatsutake* has a unique nitrogen assimilation mechanism

On the basis of our comparisons, we determined that *T. bakamatsutake* SF-Tf05 lacks *nit-6* (KO: K17877), which encodes a nitrite reductase that catalyzes the conversion of nitrite to ammonia in an assimilatory nitrate reduction pathway. Another functional homolog (*nir1/nirA*) in this pathway encodes a ferredoxin-type nitrite reductase that is present mainly in plants and bacteria. However, an ortholog of *nir1/nirA* was not identified in Tbkm_v1 or TMA_r1.0. Thus, *nit-6* may be the only nitrite reductase gene in *Tricholoma* species. Notably, *SF09g00300* in Tbkm_v1 encodes a protein similar to a nitrate/nitrite transporter identified in saprotrophic Agaricomycetes fungi, including *Cyathus striatus* (GenPept accession code KAF9007402) and *Mycena venus* (KAF7357751), as well as in many other species. In contrast, the *T. matsutake* genome, which contains *nit-6*, does not include an *SF09g00300* ortholog. Our PCR experiments demonstrated that the genomes of all 10 examined *T. bakamatsutake* isolates collected from different regions in Japan carry *SF09g00300*, but not *nit-6* (Supplementary Fig. 4a). Conversely, the genomic analysis of 12 isolates of different *Tricholoma* species (i.e. *Tricholoma anatolicum*, *T. fulvocastaneum*, *Tricholoma murrillianum*, and *T. matsutake*) revealed the presence of *nit-6* and the absence of *SF09g00300* (Supplementary Fig. 4b and c). Interestingly, *Tricholoma caligatum* and *Tricholoma mesoamericanum* apparently lack both *nit-6* and *SF09g00300* (Supplementary Fig. 4c). The analysis of the products of the PCR amplification using *nit-6* primers and *T. fulvocastaneum* strains LAOS1 (originally collected in Laos) and WK-N-1 (Japan) confirmed that the sequences did not match *nit-6*, indicative of nonspecific PCR amplifications. We speculated that the probable nitrate/nitrite transporter encoded by *SF09g00300* mediates an alternative nitrogen assimilation mechanism in *T. bakamatsutake*. Additionally, the nitrogen assimilation mechanisms of *T. caligatum* and *T. mesoamericanum* do not involve *nit-6* and *SF09g00300*. These results suggest that *T. bakamatsutake* evolved a nitrite uptake and assimilation system that has not been observed in other members of *Tricholoma* section *Caligata*.

### Chromosome-scale synteny between Tbkm_v1 and TMA_r1.0

**Fig. 4.**
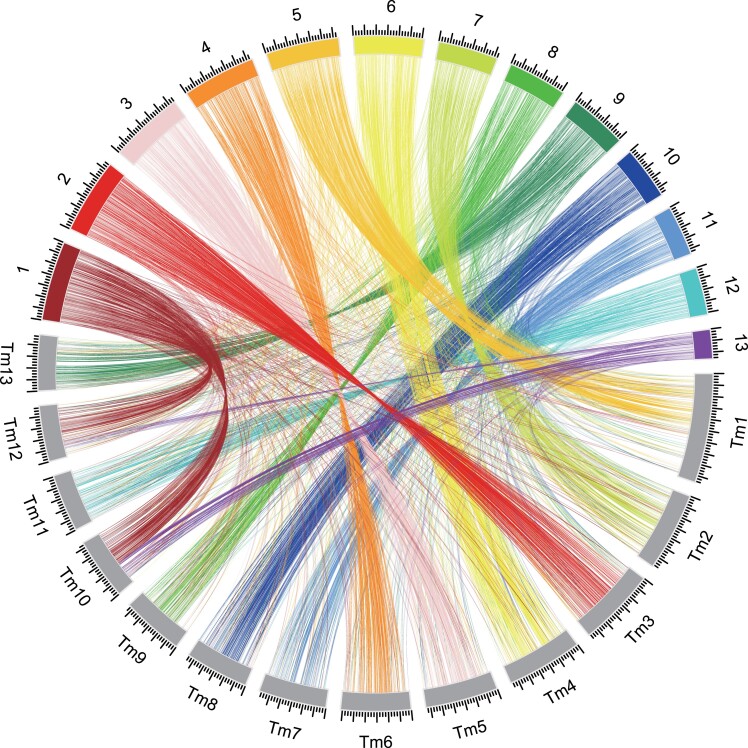
Chromosome-wide comparison of the *T. bakamatsutake* (Tbkm_v1) and *T. matsutake* (TMA_r1.0) genomes. TBLASTN searches were performed using the gene sequences located within nonrepetitive regions as queries. The searches were restricted to matches with *E*-values of 1.0e^−10^ or less. The best-hit regions were considered as the corresponding regions. The results were visualized using the Circos software.

The telomere-to-telomere assemblies of all 13 chromosomes in *T. bakamatsutake* (Tbkm_v1) and *T. matsutake* (TMA_r1.0; a total length of 161,040,721 bases) enabled us to characterize the chromosome-wide syntenic regions between the 2 genomes. Because repetitive sequences occupied more than 70% of the complete genome in both Tbkm_v1 and TMA_r1.0, simple global alignments of chromosome or genome segments were not useful. Accordingly, we used the deduced amino acid sequences of the predicted genes in nonrepetitive regions in Tbkm_v1 and performed TBLASTN searches to find the corresponding genomic regions in TMA_r1.0. Using this approach, multiple hits in different genomic loci due to dispersed repeated sequences were effectively eliminated. We identified and visualized the chromosome-wide synteny between these 2 species (Fig. 4 and Supplementary Table 3). Chromosomes 1 and 13 in Tbkm_v1 (Tb1 and Tb13) had a chimeric structure that matched approximately half of chromosomes 10 and 12 in TMA_r1.0 (Tm10 and Tm12). There was an almost 1-to-1 correspondence among the other 11 chromosomes, but the corresponding chromosomes in the 2 assemblies were not sequential, even though the 13 chromosomes were numbered according to the descending order of their lengths in both assemblies. Moreover, the examination of the corresponding regions between Tb5/Tb11 and Tm1/Tm11 revealed multiple inversions within the chromosomes. These results indicated that during species differentiation, *T. bakamatsutake* and *T. matsutake* independently accumulated different types of DNA in their chromosomes, while still maintaining the basic number of chromosomes and resembling the fruiting body shape and aroma in natural habitats (Supplementary Fig. 4). Such large alterations in chromosomal structures and multiple inversions within chromosomes may be related to the mechanisms underlying the reproductive isolation of these species.

### *Tricholoma* section *Caligata* consists of at least 3 major karyotypes

**Fig. 5.**
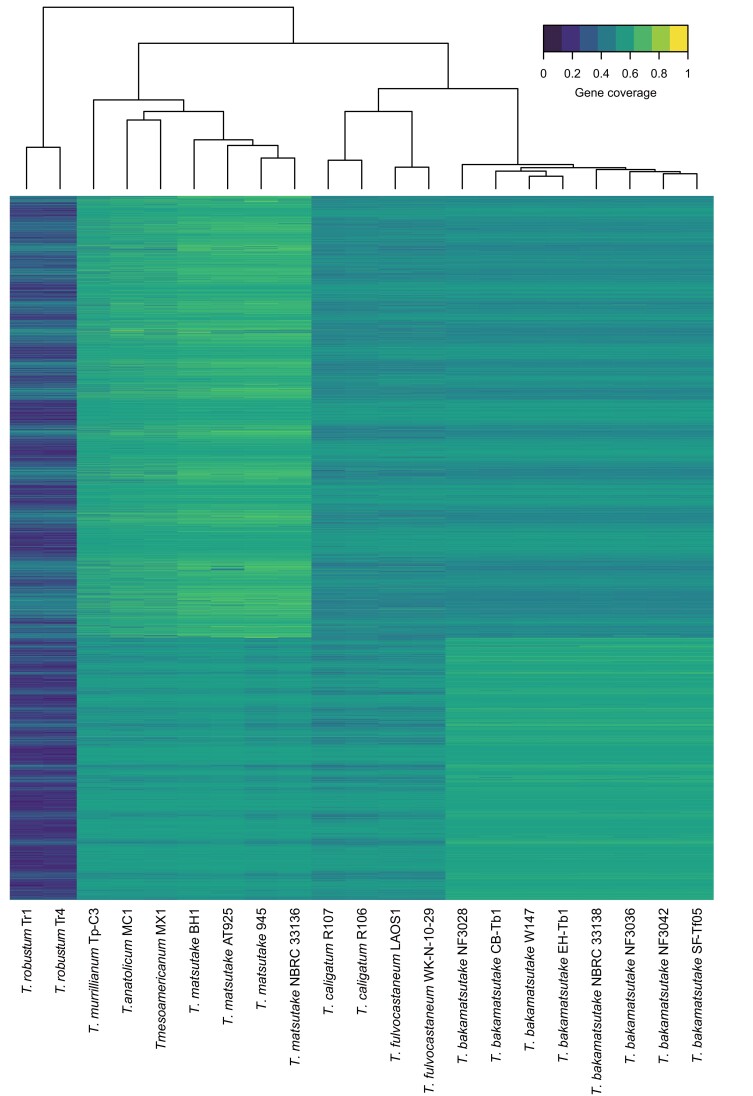
Cluster analysis of *Tricholoma* species according to the presence of shared genes. The default parameters of the heatmap function were used to cluster and visualize the percentages of the covered regions for the predicted genes in Tbkm_v1 and TMA_r1.0. The analysis revealed at least 3 groups within *Tricholoma* section *Caligata*.

The comparison of the conserved genes in Tbkm_v1 and TMA_r1.0 detected the chromosome-wide synteny in these species. We then investigated the conservation of genes among *Tricholoma* section *Caligata* species. Briefly, we performed a random shotgun sequencing analysis of the following 8 strains: *T. bakamatsutake* (SF-Tf05 and NBRC 33138), *T. matsutake* (NBRC 33138), *T. anatolicum* (MC1), *T. caligatum* (R107), *T. fulvocastaneum* (WK-N-1), *T. mesoamericanum* (MX1), and *T. murrillianum* (Tp-C3). The generated sequencing reads as well as 1 read for *T. matsutake* 945 obtained from SRA (BioProject code PRJNA200596) were mapped to the Tbkm_v1 and TMA_r1.0 sequences. The mapping rates are summarized in Supplementary Table 4. Three different groups had high (>90%) and low (approximately 50%) fractions of mapped reads in the 2 assemblies. The first group consisted of the 8 *T. bakamatsutake* strains (high and low fractions of mapped reads in Tbkm_v1 and TMA_r1.0, respectively). The second group included *T. matsutake*, *T. anatolicum*, *T. mesoamericanum*, and *T. murrillianum* (high and low fractions of mapped reads in TMA_r1.0 and Tbkm_v1, respectively). The remaining 2 species, *T. caligatum* and *T. fulvocastaneum*, formed the third group (low fractions of mapped reads in Tbkm_v1 and TMA_r1.0). These findings implied that approximately half of the genomic regions were common to all of the examined *Tricholoma* species, but the other half differentiated into at least 3 groups. We calculated the read coverage (i.e. percentage of the gene regions that were covered by mapped reads) in both assemblies and used it as the input for the cluster analysis that used Euclidean distances as the distance matrix. The cluster analysis also revealed 3 major clades (Fig. 5). In the “*matsutake*” clade, *T. anatolicum* and *T. mesoamericanum* were most similar to *T. matsutake*, followed by *T. murrillianum*. The *T. bakamatsutake* strains formed their own “*bakamatsutake*” clade, which was proximal to the third clade “*caligatum*,” which consisted of *T. caligatum* and *T. fulvocastaneum*. And these 3 clades were all distinct from the 2 *T. robustum* strains, which is consistent with the current taxonomy that places *T. robustum* in *Tricholoma* section *Megatricholoma*. These results revealed that the *Tricholoma* section *Caligata* comprises at least 3 major groups at the genome level.

### Conservation and differentiation of genes and chromosomes in *Tricholoma* section *Caligata*

As described above, our analysis showed that *T. bakamatsutake* and *T. matsutake* have the same number of chromosomes, but there are several interchromosomal and intrachromosomal rearrangements. Chromosomal rearrangements, most typically inversions, inhibit synapsis during meiosis, resulting in defective gametogenesis and/or gametophytic lethality. For example, the “balancer chromosomes” in *Drosophila melanogaster*, which were described nearly 100 years ago and have since served as an essential genetic resource for fruit fly studies (Miller *et al*. 2019), contain multiple inverted and rearranged segments and lethal markers to maintain recessive lethal mutations during natural selection. In plants, chromosomal inversions are major factors that have facilitated the evolution of reproductive isolation between populations (Baack *et al*. 2015). Accordingly, the chimeric structures and multiple inversions in *T. bakamatsutake* and *T. matsutake* chromosomes may reflect the changes during the differentiation between these 2 species.

In addition to chromosome-scale structural differences, we also found considerable diversity in the gene contexts between the 2 studied species. Our gene conservation analysis showed that more than 40% of the *T. matsutake* genes are not present in the *T. bakamatsutake* genome, whereas 83.54% of the *T. bakamatsutake* genes are also present in *T. matsutake*. These observations are consistent with the results of previous research using transposon analyses (Murata, Ota, Yamaguchi, *et al*. 2013), which indicated that *T. bakamatsutake* may have diversified earlier than many of the other species belonging to *Tricholoma* section *Caligata*, including *T. fulvocastaneum*, *T. caligatum*, *T. murrillianum*, *T. mesoamericanum*, *T. anatolicum*, and *T. matsutake*. The diversification of *T. matsutake* may have occurred most recently. A DNA barcode analysis showed that *T. bakamatsutake* is relatively closely related to *T. matsutake* (Murata, Ota, Yamada, *et al*. 2013). Our gene conservation and read-mapping analyses indicated that the genomic structures and gene contexts of *T. caligatum* and *T. fulvocastaneum* differ from those of *T. bakamatsutake* and *T. matsutake*. This is consistent with the results of a recent phylogenetic analysis based on internal transcribed spacer (ITS) sequences (Herrera *et al*. 2022). Thus, *T. caligatum* and its related species form an independent group in the *Tricholoma* section *Caligata*. Phylogenetic analysis based on ITS sequences revealed that *Tricholoma dulciolens* and *Tricholoma ilkkae* belong to the same clade as *T. bakamatsutake*, *T. fulvocastaneum*, and *T. caligatum* (Aoki *et al*. 2022; Herrera *et al*. 2022). There is considerable confusion in the taxonomy of *Tricholoma* species. It has been confirmed that *T. dulciolens* is morphologically and phylogenetically distinct from *T. caligatum* (Kytövuori 1988; Murata, Ota, Yamada, *et al*. 2013). Although we did not analyze *T. dulciolens* and *T. ilkkae* in the present study, we speculate that these species form an independent clade. Therefore, the *Tricholoma* section *Caligata* likely comprises 4 major karyotypes.

In Ascomycota (e.g. *Neurospora crassa*), centromeric regions consist of degenerate copies of retrotransposons and simple sequence repeats (Smith *et al*. 2012). Furthermore, LINEs are highly enriched near the center of chromosomes (i.e. centromeric regions) in *T. matsutake*, but a similar localization of general LINEs was not observed in *T. bakamatsutake*, with the exception of a few chromosomes. Instead, at least 10 of the 13 *T. bakamatsutake* chromosomes had *marY2N*-rich regions, often near the center, but sometimes at 1 end or at multiple locations where the *marY2N* content exceeded 50% of the analyzed interval (5 kb). An experimental approach, such as ChIP-seq with CenH3, may be necessary to further demonstrate the link between *marY2N* and the centromere function, which has not been determined for any *Tricholoma* species.

## Supplementary Material

jkad198_Supplementary_Data

## Data Availability

The high-throughput sequencing data used in the present study have been deposited in the Sequence Read Archive under BioProject code PRJNA914084. The assembled chromosomal and mitochondrial genomic sequences were deposited in GenBank with the accession codes CP114857–CP114870. The source codes of the programs are available on GitHub (https://github.com/hirnc/tbkm). The annotation of the Tbkm_v1 genome assembly (Supplementary File 1) and other Supplementary materials are available at G3 online. Supplemental material available at G3 online.
