# Supplementary material for: Near-complete de novo assembly of Tricholoma bakamatsutake chromosomes revealed the structural divergence and differentiation of Tricholoma genomes
Source: G3 (Bethesda). 2023 Sep 2;13(11):jkad198. doi: 10.1093/g3journal/jkad198 (PMC10627285; doi:10.1093/g3journal/jkad198)
Supplement: jkad198_Supplementary_Data [file jkad198_supplementary_data.zip › Supplemental_Figures_G3-2023-404452.pdf]

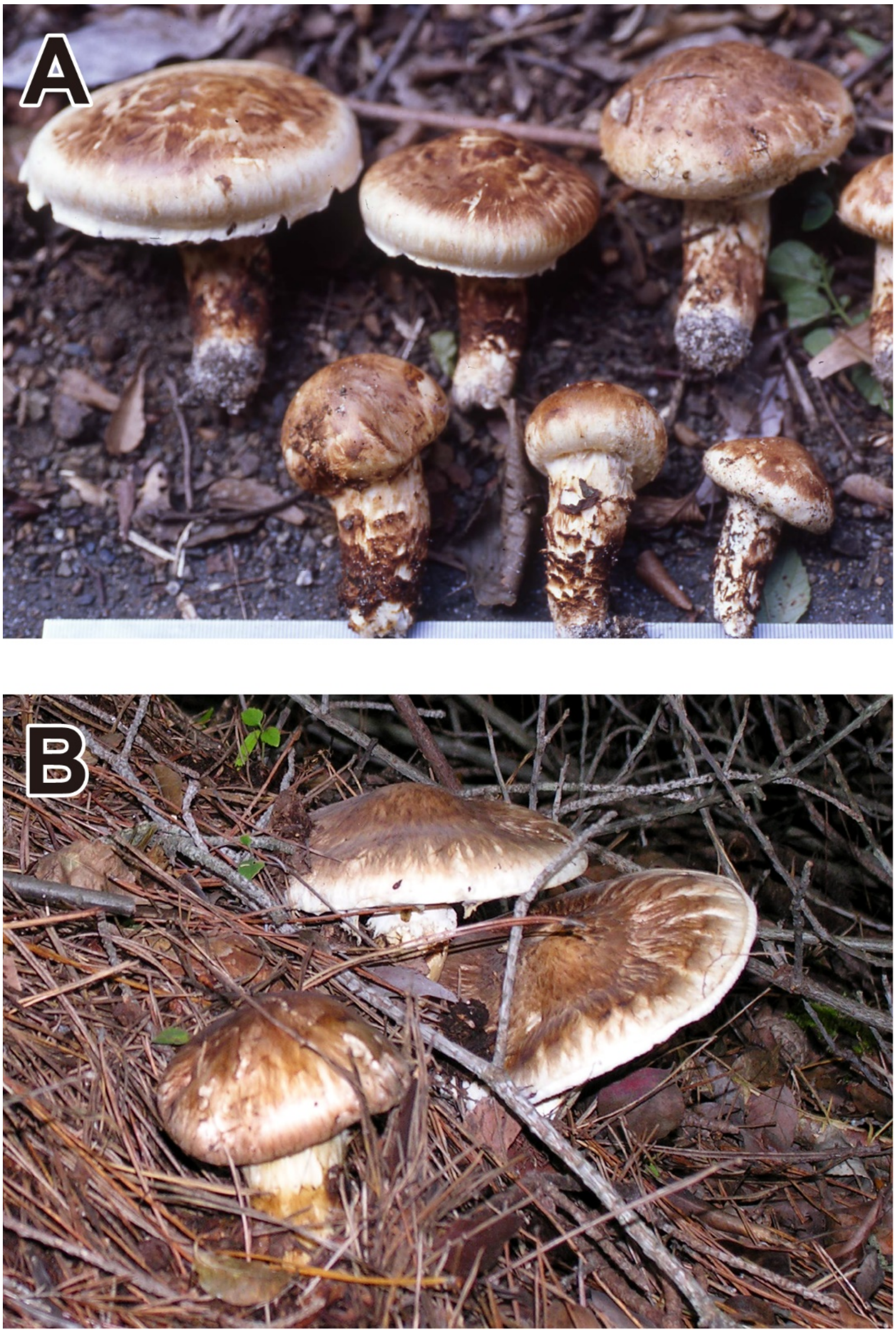

Fig. S1. Fruiting bodies of (A) *T. bakamatsutake* and (B) *T. matsutake* in natural habitats.

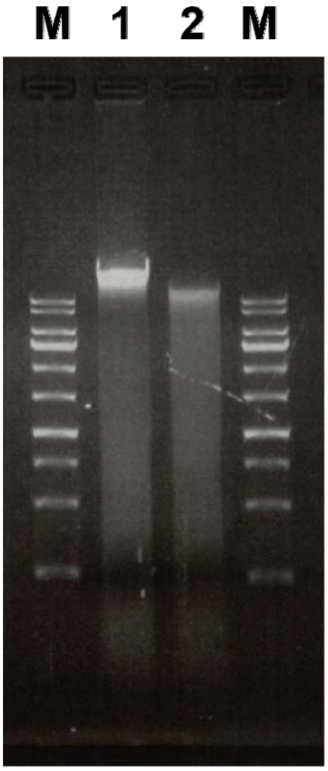

Fig. S2. Agarose gel electrophoresis of purified genomic DNA from *T. bakamatsutake* SF-Tf05.

M: 1 kb ladder (APRO science, KE-2510); lane 1: genomic DNA purified from *T. bakamatsutake* SF-Tf05 (undigested, 500 ng); lane 2: genomic DNA purified from *T. bakamatsutake* SF-Tf05, digested with EcoRI (500 ng).

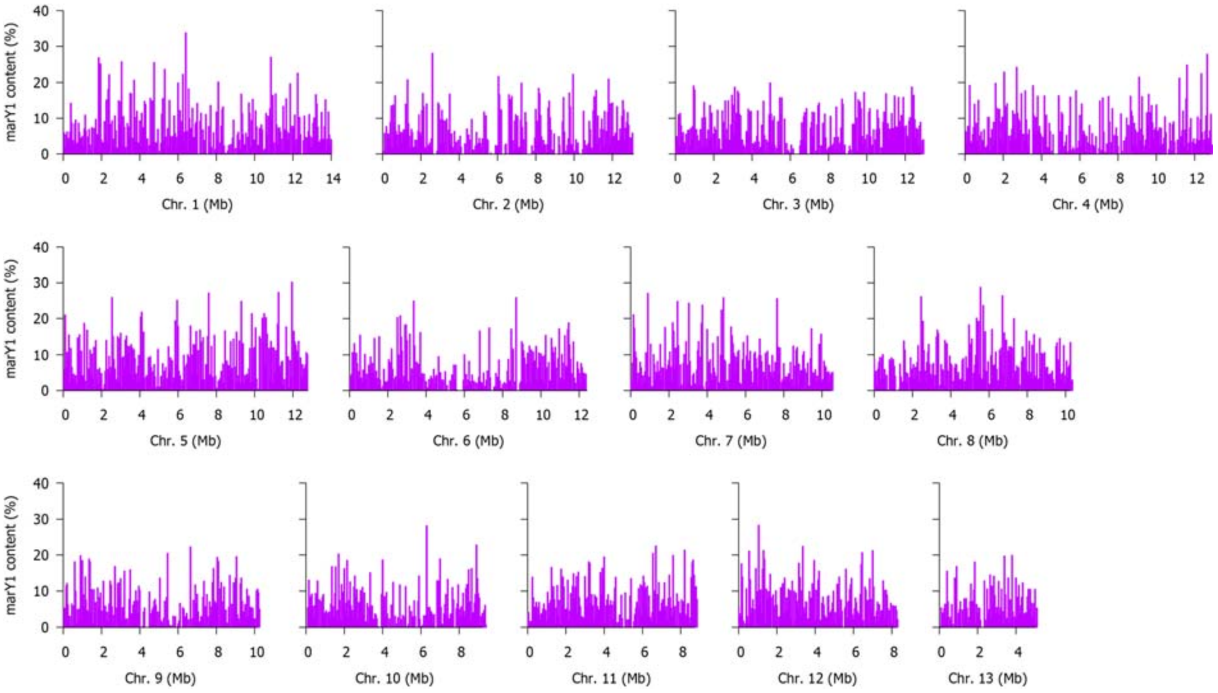

Fig. S3. *marY1* contents in the 13 *T. bakamatsutake* SF-Tf05 chromosomes.

The *marY1* contents among different chromosomes were determined using a window size of 50 kb. The results indicate that *marY1* is distributed throughout the genome.

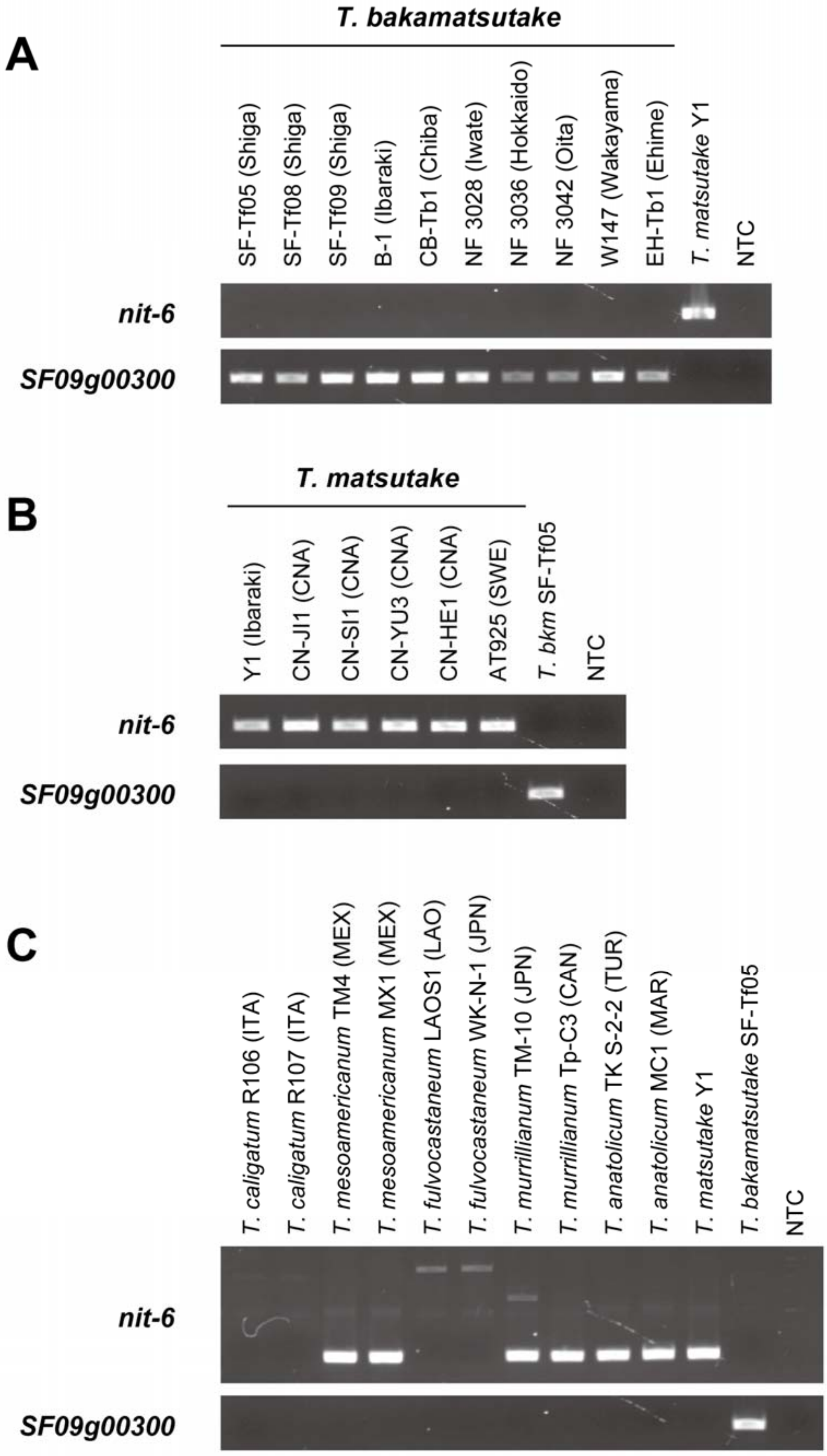
A
T. bakamatsutake
SF-Tf05 (Shiga)
SF-Tf08 (Shiga)
SF-Tf09 (Shiga)
B-1 (Ibaraki)
CB-Tb1 (Chiba)
NF 3028 (Iwate)
NF 3036 (Hokkaido)
NF 3042 (Oita)
W147 (Wakayama)
EH-Tb1 (Ehime)
T. matsutake Y1
NTC
nit-6
SF09g00300
B
T. matsutake
Y1 (Ibaraki)
CN-JI1 (CNA)
CN-SI1 (CNA)
CN-YU3 (CNA)
CN-HE1 (CNA)
AT925 (SWE)
T. bkm SF-Tf05
NTC
nit-6
SF09g00300
C
T. caligatum R106 (ITA)
T. caligatum R107 (ITA)
T. mesoamericanum TM4 (MEX)
T. mesoamericanum MX1 (MEX)
T. fulvocastaneum LAOS1 (LAO)
T. fulvocastaneum WK-N-1 (JPN)
T. murrillianum TM-10 (JPN)
T. murrillianum Tp-C3 (CAN)
T. anatolicum TK S-2-2 (TUR)
T. anatolicum MC1 (MAR)
T. matsutake Y1
T. bakamatsutake SF-Tf05
NTC
nit-6
SF09g00300

Fig. S4. Distribution of *nit-6* and *SF09g00300* in *T. bakamatsutake* and its allied species.

The presence and absence of *nit-6* and *SF09g00300*, encoding a nitrite reductase and a nitrate/nitrite transporter, respectively, in (A) *T. bakamatsutake*, (B) *T. matsutake*, and (C) other *Tricholoma* section *Caligata* species were determined on the basis of a PCR analysis. The geographical locations where the strains were originally collected are indicated in parentheses. The prefectures in Japan are provided in full, whereas three-letter codes are provided for countries (ISO 3166-1 alpha-3).
