# Supplementary material for: Near-complete de novo assembly of Tricholoma bakamatsutake chromosomes revealed the structural divergence and differentiation of Tricholoma genomes
Source: G3 (Bethesda). 2023 Sep 2;13(11):jkad198. doi: 10.1093/g3journal/jkad198 (PMC10627285; doi:10.1093/g3journal/jkad198)
Supplement: jkad198_Supplementary_Data [file jkad198_supplementary_data.zip › Supplemental_Tables_G3-2023-404452.pdf]

Table S1. Genetic statistics for the *T. bakamatsutake* SF-Tf05 chromosomes

**Chromosome**	Length (bp)	GC (%)	Number of genes		GenBank accession
			Protein coding	tRNA	
**1**	14,073,764	43.79	1,071	26	CP114857
**2**	13,278,086	44.52	1,342	29	CP114858
**3**	13,010,463	44.10	1,125	35	CP114859
**4**	12,983,161	44.34	1,203	31	CP114860
**5**	12,957,022	43.72	877	65	CP114861
**6**	12,427,164	44.34	1,104	34	CP114862
**7**	10,612,962	43.39	636	30	CP114863
**8**	10,437,183	43.41	668	20	CP114864
**9**	10,325,240	44.13	758	36	CP114865
**10**	9,419,585	44.14	783	13	CP114866
**11**	8,909,905	43.68	600	16	CP114867
**12**	8,408,117	43.45	517	25	CP114868
**13**	5,225,559	43.70	376	12	CP114869
**Total**	**142,068,211**	**43.94**	**11,060**	**372**	—

Table S2. Categorization of the predicted genes in Tbkm_v1 according to their functions

KEGG Orthology (KO) entry and functional category		No. of genes
ko04131	Membrane trafficking	411
ko02000	Transporters	209
ko03019	Messenger RNA biogenesis	204
ko03036	Chromosome and associated proteins	197
ko03009	Ribosome biogenesis	180
ko03021	Transcription machinery	152
ko01002	Peptidases and inhibitors	149
ko04121	Ubiquitin system	136
ko03041	Spliceosome	131
ko03011	Ribosome	128
ko03029	Mitochondrial biogenesis	127
ko01001	Protein kinases	119
ko03400	DNA repair and recombination proteins	96
ko01009	Protein phosphatases and associated proteins	95
ko03032	DNA replication proteins	80
ko03110	Chaperones and folding catalysts	78
ko03016	Transfer RNA biogenesis	76
ko03000	Transcription factors	67
ko00190	Oxidative phosphorylation	66
ko03012	Translation factors	59
ko04147	Exosome	59
ko01007	Amino acid related enzymes	53
ko01003	Glycosyltransferases	52
ko01004	Lipid biosynthesis proteins	31
ko03051	Proteasome	31
ko04812	Cytoskeleton proteins	31
ko00010	Glycolysis / Gluconeogenesis	30

Table S3. Corresponding chromosomes between *T. bakamatsutake* (Tbkm_v1) and *T. matsutake* (TMA_r1.0)

T. bakamatsutake chromosome (Tbkm_v1)	*T. matsutake* chromosome (TMA_r1.0)
1	10 and 12
2	3
3	5
4	6
5	1 *
6	4
7	2
8	9
9	13
10	8
11	7
12	11 *
13	10 and 12

* Probable intra-chromosomal inversions were detected

Table S4. Percentage of the reads that were mapped to the *T. bakamatsutake* and *T. matsutake* reference genome sequences

Strain and reference genome	*T. bakamatsutake* (Tbkm_v1) %	*T. matsutake* (TMA_r1.0) %
**"Bakamatsutake" group**		
T. bakamatsutake SF-Tf05	**99.21**	60.02
T. bakamatsutake NBRC33138	**97.00**	58.77
T. bakamatsutake CB-Tb1	**98.26**	57.25
T. bakamatsutake EH-Tb1	**98.77**	57.65
T. bakamatsutake NF3028	**97.81**	57.67
T. bakamatsutake NF3036	**98.07**	58.65
T. bakamatsutake NF3042	**98.31**	58.32
T. bakamatsutake P752	**98.58**	58.61
T. bakamatsutake W147	**98.30**	57.87
**"Matsutake" group**		
T. matsutake 945	29.70	**98.57**
T. matsutake NBRC33138	51.61	**96.17**
T. matsutake AT925	52.58	**98.05**
T. matsutake BH1	53.69	**98.65**
T. anatolicum MC1	56.80	**95.54**
T. mesoamericanum MX1	51.26	**96.19**
T. murrillianum Tp-C3	54.41	**93.37**
**"Caligatum" group**		
T. caligatum R106	56.47	65.47
T. caligatum R107	57.22	66.28
T. fulvocastaneum LAOS1	38.97	46.23
T. fulvocastaneum WK-N-1	40.55	48.32
**Outgroup (Section Megatricholoma*)**		
T. robustum Tr1	16.09	25.11
T. robustum Tr4	15.38	25.12

* Heilmann-Clause (2017)
